# Membrane properties modulate methane oxidation by particulate methane monooxygenase

**DOI:** 10.1016/j.jbc.2026.113435

**Published:** 2026-08-12

**Authors:** Callie G. Miller, Frank J. Tucci, Genevieve R. Nemeth, Sergey Stolyar, Mary E. Lidstrom, Amy C. Rosenzweig

**Affiliations:** 1 Departments of Molecular Biosciences and of Chemistry, Northwestern University, Evanston, Illinois, USA; 2 Departments of Chemical Engineering and of Microbiology, University of Washington, Seattle, Washington, USA

**Keywords:** copper monooxygenase, metalloenzyme, membrane enzyme, lipid bilayer, cryoelectron microscopy, cryoelectron tomography, methane oxidation, ammonia oxidation

## Abstract

The copper-dependent membrane monooxygenases particulate methane monooxygenase (pMMO) and ammonia monooxygenase (AMO) oxidize methane to methanol and ammonia to hydroxylamine, respectively. These enzymes, which are important targets for biotechnology, reside in intracytoplasmic membranes (ICMs) where they form densely packed hexagonal arrays. While cryoEM structures of pMMO and AMO in ICMs have revealed closely associated lipids, little is known about how specific lipids and membrane morphologies influence activity. Here we show through cryoelectron tomography (cryoET) that three species of methane- and ammonia-oxidizing bacteria exhibit different types of ICM ultrastructure. Reconstitution of *Methylococcus capsulatus* (Bath) pMMO into liposomes replicated the array structure, allowing a systematic dissection of how liposome diameter and composition affect activity. Proteoliposome activity is inversely correlated with liposome size, suggesting that pMMO activity may be higher in membranes with increased surface curvature. Further, a comparison of lipids isolated from methanotrophs (native lipids), phosphatidylcholine (PC), and phosphoethanolamine (PE) showed that PE confers increased activity, with maximal activity observed for unsaturated PEs. Methane solubility measurements indicate that these enhancements are specific to pMMO. Cardiolipin further increases activity, consistent with its enrichment in *M. capsulatus* (Bath) cells. To assess pMMO-pMMO interactions in the ICMs, a 6 Å resolution cryoelectron microscopy (cryoEM) structure of three neighboring pMMO trimers was determined, revealing their arrangement in the array as well as specific residues and lipids mediating interaction interfaces. Taken together, these findings provide insight into the impact of the membrane environment on pMMO function and establish a platform for examining pMMOs and AMOs in tunable lipid environments.

Methane is a potent greenhouse gas that contributes significantly to global warming (1). As a major biological sink for methane emissions (2), methanotrophic bacteria have been the focus of intense efforts to couple methane mitigation with production of value-added chemicals (3). Aerobic methanotrophs convert methane to methanol using methane monooxygenases (MMOs) (4). Almost all methanotrophs use the copper-dependent, particulate MMO (pMMO), while a subset of strains expresses the iron-dependent, soluble MMO (sMMO) under conditions of copper limitation (4). As the MMO with the higher affinity for methane, pMMO is a prime candidate for methane removal from air (5, 6). pMMO comprises ∼80% of the total protein in methanotroph membranes (7), and is housed in intracytoplasmic membranes (ICMs), the formation of which is triggered by copper (8, 9). The ICMs span the interior of the cell with varying architectures: type I gammaproteobacterial methanotrophs like *Methylococcus capsulatus* (Bath) form vesicular disc-shaped membranes perpendicular to the concentric outer and cytoplasmic membranes, whereas type II alphaproteobacterial methanotrophs like *Methylocystis* sp. Rockwell exhibit concentric paired membranes around the cell periphery (10). Notably, the pMMO homolog ammonia monooxygenase (AMO), which converts ammonia to hydroxylamine in ammonia-oxidizing bacteria, also resides in ICMs (11, 12).

While pMMO research has long focused on elucidating the molecular details of the copper active site (4, 13), it was not until the enzyme was structurally characterized in lipid nanodiscs and native membranes that the site currently believed to be the catalytic center, Cu_D_, was identified (12, 14, 15). The Cu_D_ site was not observed in detergent-solubilized samples, which exhibit little to no enzymatic activity (16). The highest pMMO activity is measured for whole cells, followed by isolated membrane fractions, and then pMMO reconstituted into lipid nanodiscs and bicelles (4, 14, 17, 18). These observations underscore the importance of the membrane environment for activity. Moreover, within the ICMs, pMMO, as well as AMO, forms tightly packed hexagonal arrays that have been linked to activity (19). In support of these arrays promoting pMMO activity, methane oxidation is reduced in nanodiscs and bicelles, which accommodate only a single pMMO trimer, as compared to isolated membranes with intact pMMO hexagonal arrays.

Despite the functional importance of the ICMs and the arrangement of pMMO therein, virtually nothing is known about how the physicochemical properties of the membrane influence pMMO activity. In nanodiscs, lipids isolated from *M. capsulatus* (Bath) confer increased activity over synthetic lipids such as 1,2-dimyristoyl-sn-glycero-3-phosphocholine (DMPC) and 1-palmitoyl-2-oleoylphosphatidylcholine (POPC) (14). Lipidomic analysis of *M. capsulatus* (Bath) cells revealed the presence of 49% phosphatidylethanolamine (PE), 22% phosphatidylglycerol (PG), 9% phosphatidylcholine (PC), and 14% cardiolipin (CL) and CL precursors, along with 6% unidentified lipids (12, 14). CL is similarly enriched in mitochondrial inner membranes, where it is essential for the assembly and activity of respiratory complexes (20, 21), and could play an analogous role in pMMO function. Additionally, the abundance of conical PE lipids may be significant as they enforce negative membrane curvature (22), although the degree of curvature in ICMs has not been quantified. Cryoelectron microscopy (cryoEM) maps of pMMO and AMO in their native membranes show well-defined lipid densities both on the periphery and in the central pore of the trimer, but the heterogeneous native membrane lipid content precluded assigning identities to these lipids, which were therefore modeled simply as PE (12). How different membrane morphologies and specific lipids impact pMMO function thus remains a major unanswered question.

Here we have begun to address the relationship between pMMO/AMO function and membrane environment via cryoelectron tomography (cryoET) of methanotroph and ammonia oxidizer cells, activity studies of pMMO in proteoliposomes, and cryoEM of pMMO arrays. Proteoliposomes circumvent the heterogeneity of native membranes and the inability to recapitulate arrays in nanodiscs or bicelles, providing a membrane mimetic system that allows for systematic investigation of membrane properties. The results show that pMMO reforms hexagonal arrays in reconstituted liposomes, that liposome diameter impacts pMMO activity, and that liposome composition plays a key role in activity. These observations, combined with the pMMO-pMMO interfaces observed by cryoEM, represent a significant first step toward understanding how membrane properties modulate pMMO activity.

## Results and discussion

### ICMs exhibit diverse ultrastructure

**Figure 1:**
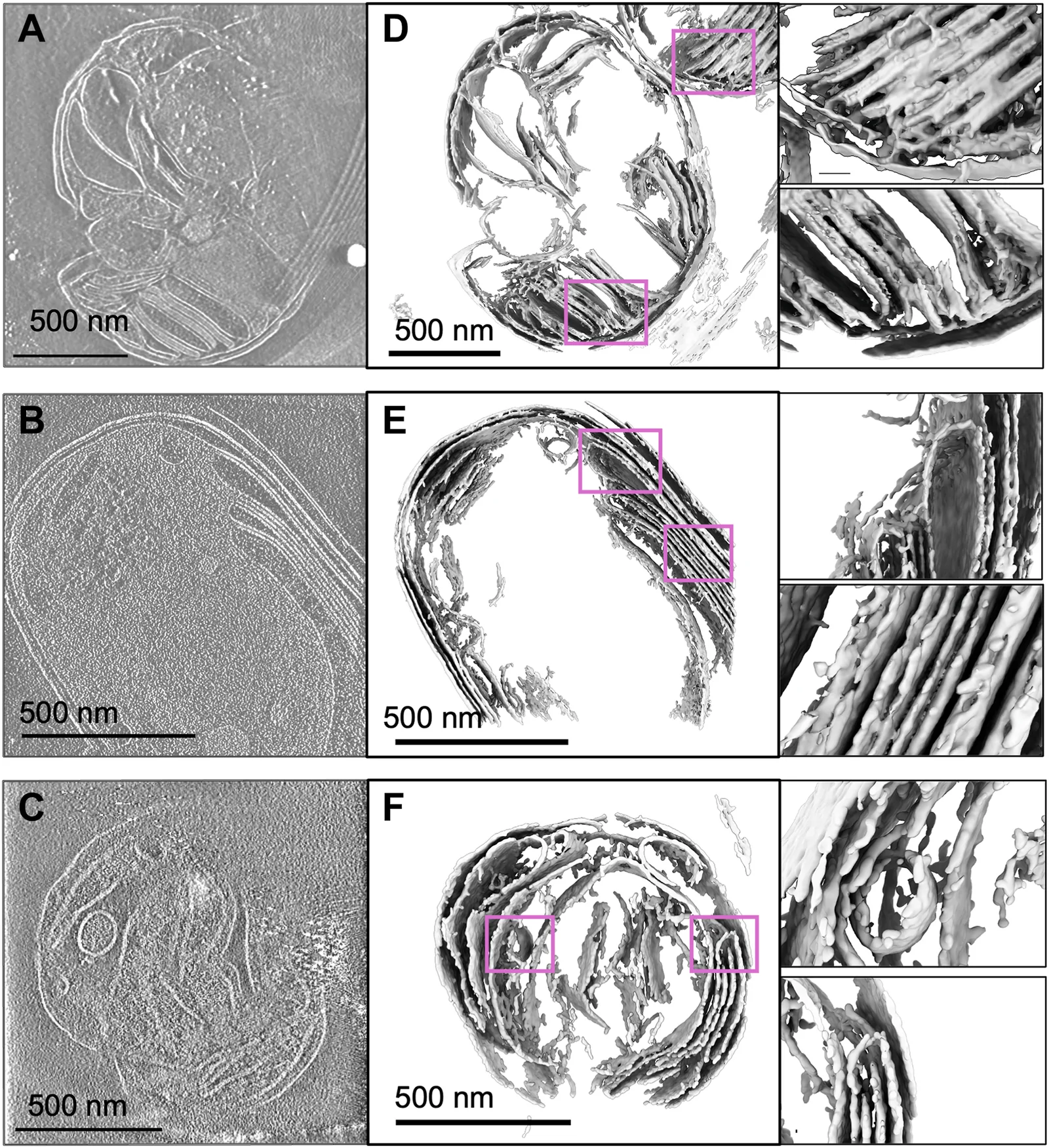
**Ultrastructure of methanotroph and ammonia oxidizer ICMs.** Tomogram slices (*left panels*) of *A*, *M. capsulatus* (Bath); *B*, *M.* sp. Rockwell; and *C*, *N. europaea* with accompanying reconstructed 3D segmentations (*D*–*F*, with regions in *pink* boxes magnified in insets). *D*, In *M. capsulatus* (Bath), the ICMs are perpendicular to the outer membrane with both highly planar and highly curved regions (insets). *E*, The concentric ICMs in *M*. sp. Rockwell also exhibit curved and planar regions (insets). *F*, The *N. europaea* ICMs are concentric with individual vesicles and overlapping regions (insets).

CryoET of cryofocused ion beam (FIB) milled lamellae from *M. capsulatus* (Bath), *M.* sp. Rockwell, and *Nitrosomonas europaea* cells revealed extensive ICMs (Figs. 1 and S1). The tomogram slices show two distinct patterns of ICMs. In the gammaproteobacterial methanotroph *M. capsulatus* (Bath), the ICMs are predominantly perpendicular to the outer membrane, as observed previously by EM (9, 10) and cryoET (19) (Fig. 1, *A* and *D*). Conversely, the ICMs of the alphaproteobacterial methanotroph *M.* sp. Rockwell and the betaproteobacterial ammonia oxidizer *N. europaea* form concentric layers, with the ICMs essentially parallel to the outer membrane (Fig. 1, *B*, *C, E*, and *F*), similar to what has been observed for other alphaproteobacterial methanotrophs, including *Methylosinus* and other *Methylocystis* species (8, 10, 23). For all three samples, regions of connectivity to the cytoplasmic membrane are visible, supporting formation via invagination (19, 24, 25), although the basis for the different morphologies is unknown. Segmentation also reveals striking details of the ICM ultrastructures. Within species and even within individual cells, there are regions of extreme membrane curvature as well as extended, almost planar membrane surfaces (Fig. 1, *D* and *E*). In addition, apparent membrane-enclosed vesicles are present (Fig. 1*F*). Such features were consistent among all cells examined (Fig. S1). These distinct membrane landscapes may differ in their lipid compositions, biophysical properties, or abilities to house pMMO/AMO arrays.

### Reconstitution and activity of pMMO liposomes

The presence of both planar and curved regions in the native methanotroph ICMs (Fig. 1) raises the possibility that membrane curvature influences pMMO activity, as has been observed for a range of enzymes and signaling proteins (26, 27, 28, 29). In the ICM-forming photosynthetic bacteria *Rhodobacter sphaeroides*, LH2 complexes induce and are localized in regions of membrane curvature (30, 31). pMMO has been suggested to initiate ICM formation (31), but assessing its relative abundance in curved and planar regions is not straightforward. However, the effects of curvature on activity can be investigated systematically using liposomes, which can be prepared at different diameters, correlating inversely with surface curvature (32). Liposomes, which differ markedly from the planar regions of the native membrane and a planar lipid bilayer, mimic the abundant curved regions of the ICMs.

pMMO proteoliposomes were prepared by either direct extrusion of native ICMs (native liposomes) or by detergent solubilization of pMMO followed by reconstitution into liposomes containing native or synthetic lipids (reconstituted liposomes) (33, 34, 35). pMMO from *M. capsulatus* (Bath) was used for these experiments because it exhibits significantly higher activity than *M.* sp. Rockwell pMMO (36). The direct-extrusion method preserves pMMO arrays within the native microenvironment without the need for detergent solubilization, but reconstitution allows for greater control over liposome composition and size. Treatment of the proteoliposomes with trypsin revealed complete degradation of the PmoB subunit, indicating that its periplasmic regions are uniformly oriented towards the outside of the liposome (Fig. S2).

**Figure 2:**
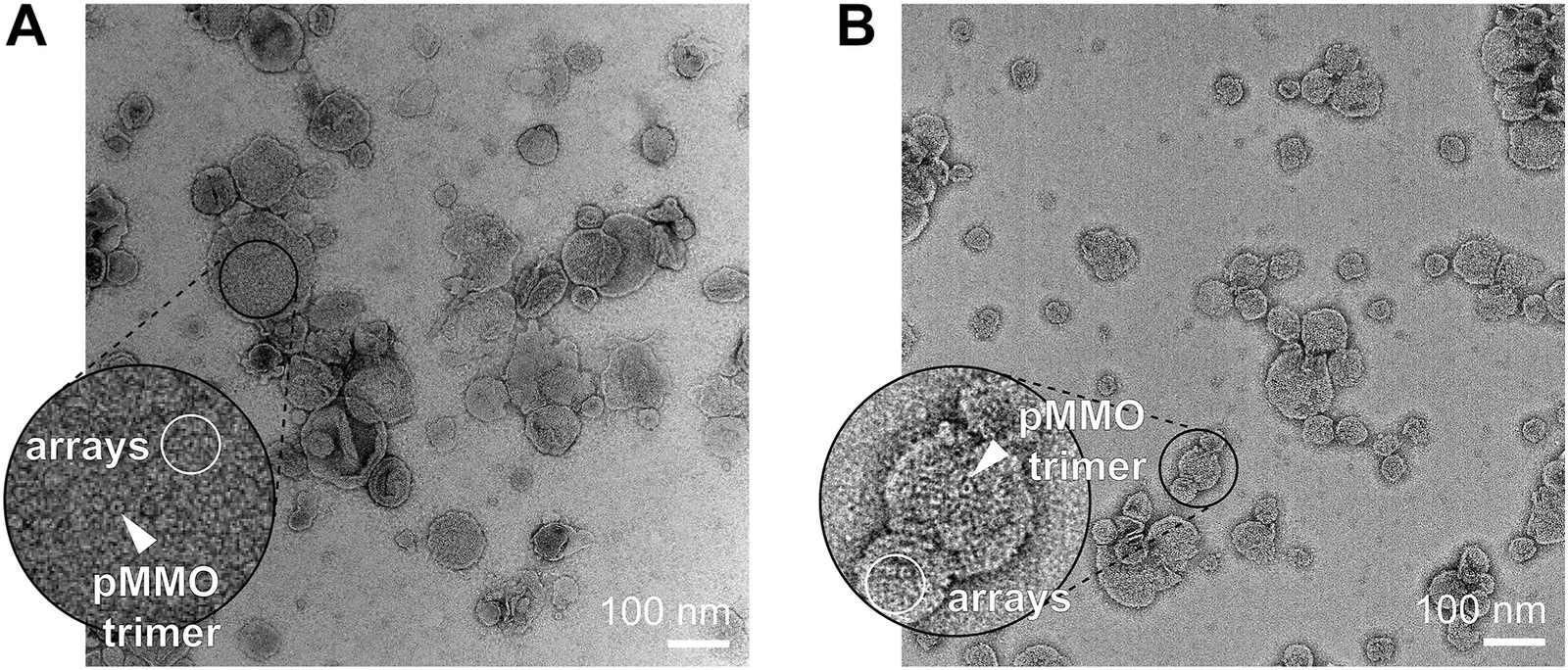
**pMMO arrays are preserved in liposomes.***A*, negative stain micrograph of liposomes directly extruded from *M. capsulatus* (Bath) membranes. *B*, negative stain micrograph of detergent-solubilized pMMO reconstituted into native lipid liposomes. Both samples were extruded at 100 nm. Zoom insets show clear areas where both the pMMO trimers and arrays are visible in both directly extruded and reconstituted liposomes.

Liposome size is dictated by the size of pores in the polycarbonate filter used for extrusion (see Experimental procedures). Dynamic light scattering (DLS) analysis of native liposomes extruded using 400 nm, 100 nm and 50 nm pore sizes gave diameters of 231 ± 114 nm, 127 ± 48, and 100 ± 4 (average of 10 measurements each), respectively, as compared to 276 ± 142 nm for unextruded membranes (Table S1). Such variability is typical (37, 38). Importantly, the pMMO arrays are preserved in the native liposomes (Fig. 2*A*), as well as in the reconstituted liposomes (Figs. 2*B* and S3), as was observed previously for partially reconstituted nanodisc preparations (19). Overall, the liposomes exhibit consistent morphologies, even for non-lamellar phosphoethanolamine (PE) lipids, which can form membrane mimetic bilayers in the presence of proteins and other lipids (22, 26, 27). In the case of pMMO, its dense packing may allow for stable bilayer formation, relieving the curvature elastic stress of a pure PE liposome. The presence of arrays in all preparations, as observed by negative stain imaging, indicates that pMMO is the predominant component of each liposome (Fig. S2). To estimate the pMMO incorporated into each liposome, protein and lipid concentrations were measured for liposomes after ultracentrifugation to remove unincorporated proteins, yielding lipid:pMMO ratios of 120:1 to 180:1 (Fig. S4). In addition to pMMO, proteomic analysis of reconstituted liposomes with POPE lipids indicated the presence of numerous other proteins in the liposome solutions (File S1).

**Figure 3:**
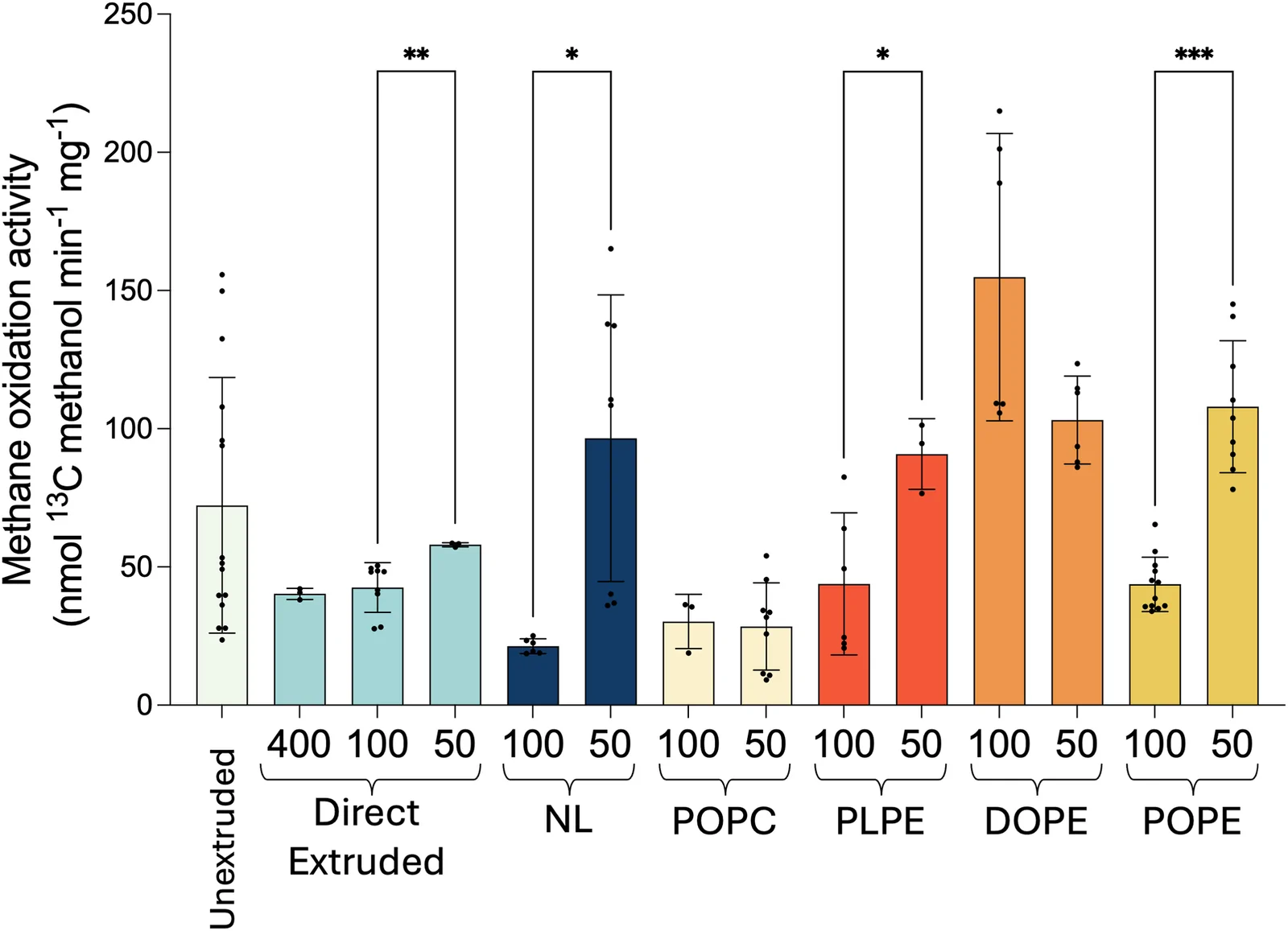
**Methane oxidation activity as a function of liposome size and composition.** Activity was measured for unextruded membranes (n = 15) and direct-extruded native liposomes at 400 (n = 3), 100 (n = 9), and 50 nm (n = 3) as well as detergent-solubilized and reconstituted liposomes with NL (n = 6, n = 8), POPC (n = 3, n = 9), PLPE (n = 6, n = 3), DOPE (n = 6, n = 6), and POPE (n = 3, n = 9), extruded at 100 nm and 50 nm (respectively to replicates). Significant values are as follows, determined by one-way ANOVA: ∗ = *p* < 0.05, ∗∗ = *p* < 0.01, ∗∗∗ = *p* < 0.001. Values with standard deviation and technical replicates are also shown in Table S2.

The different-sized native liposomes were tested for activity by monitoring conversion of ^13^CH_4_ to ^13^CH_3_OH via gas chromatography-mass spectrometry (GC-MS) (17). Prior to extrusion, native membranes exhibit a wide range of activities across different growths and membrane preparations, ranging from 25 to 150 nmol ^13^CH_3_OH min^−1^ mg^−1^ (n = 15) (Fig. 3). To control for this variability, the same cell growth and membrane preparations were used for technical replicates of each liposome type. Biological replicates for each sample obtained using different cell preparations consistently showed the same trends in activity. Native liposomes extruded using 400 nm, 100 nm, and 50 nm pore-size polycarbonate filters had average activities of 40, 43, and 58 nmol ^13^CH_3_OH min^−1^ mg^−1^, respectively (Fig. 3 and Table S2).

**Figure 4:**
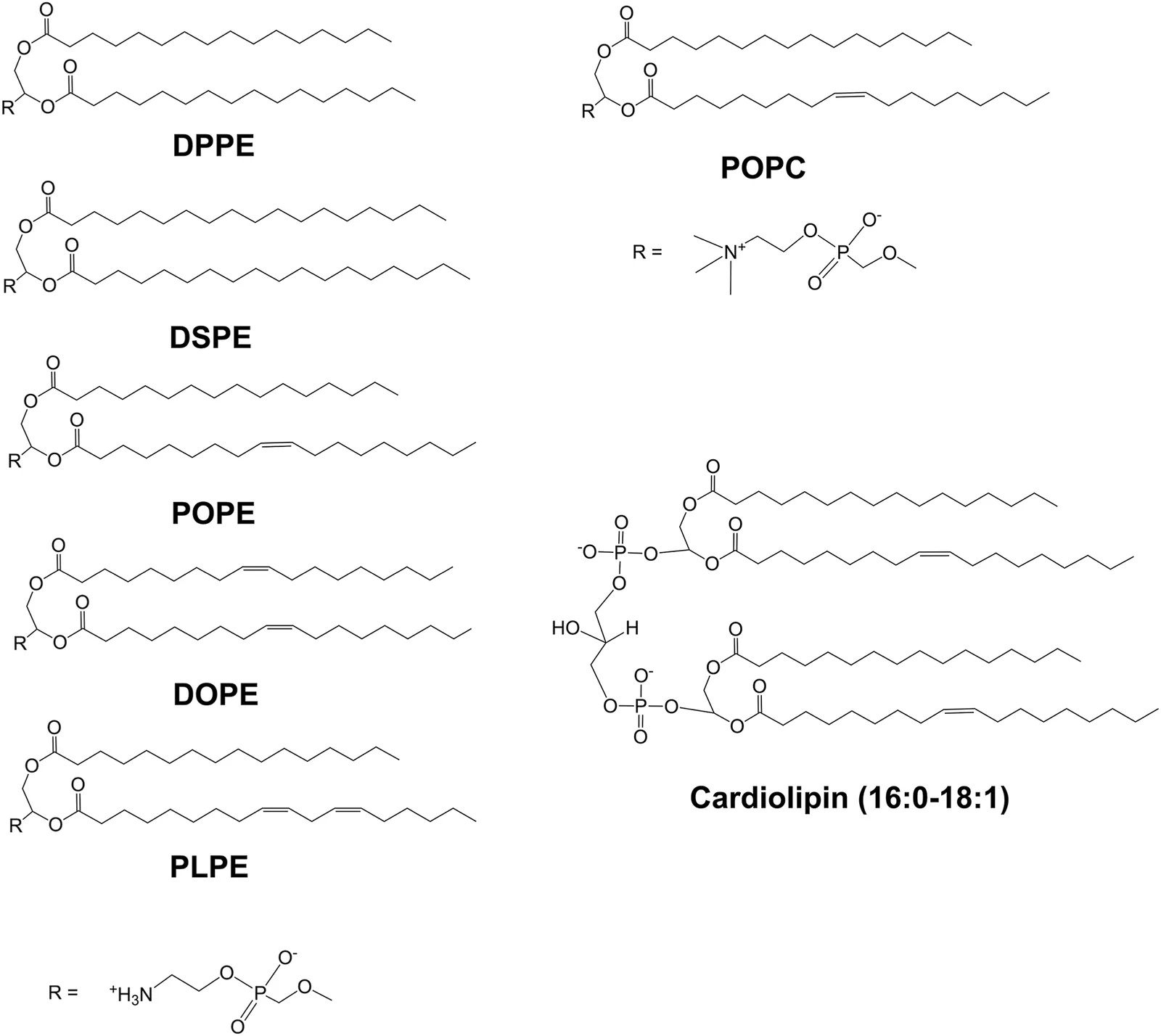
**Structures of synthetic lipids used in reconstituted liposomes**.

Reconstituted liposomes were then utilized to test the activity at different diameters. As it is challenging to separate the effects of lipid composition from those of liposome size, we compared two different extrusion sizes for each lipid composition. A panel of lipids (Fig. 4), including native lipids (NL) isolated from *M. capsulatus* (Bath) cells, were extruded at 50 nm and 100 nm, and reconstituted with detergent-solubilized pMMO using a 5:1 lipid:pMMO mol ratio. For NL, 1-palmitoyl-2-linoleoyl-sn-glycero-3-phosphoethanolamine (PLPE), and 1-palmitoyl-2-oleoyl-sn-glycero-3-phosphoethanolamine (POPE), the 50 nm reconstituted liposomes exhibited significantly higher activity than that of 100 nm reconstituted liposomes, consistent with the direct-extruded native liposomes (Fig. 3). By contrast, the liposome diameter did not significantly affect the activity of pMMO reconstituted into 1-palmitoyl-2-oleoylphosphatidylcholine (POPC) and 1,2-dioleoyl-sn-glycero-3-phosphoethanolamine (DOPE) liposomes. Notably, reconstitution into synthetic liposomes containing POPE and DOPE resulted in increased activity compared to the direct-extruded native liposomes. In addition, PLPE, DOPE, and POPE liposomes outperformed POPC. This observation is consistent with the presence of 49% PE and just 9% PC in *M. capsulatus* (Bath) cells (12, 14).

### Lipid composition affects pMMO activity

**Figure 5:**
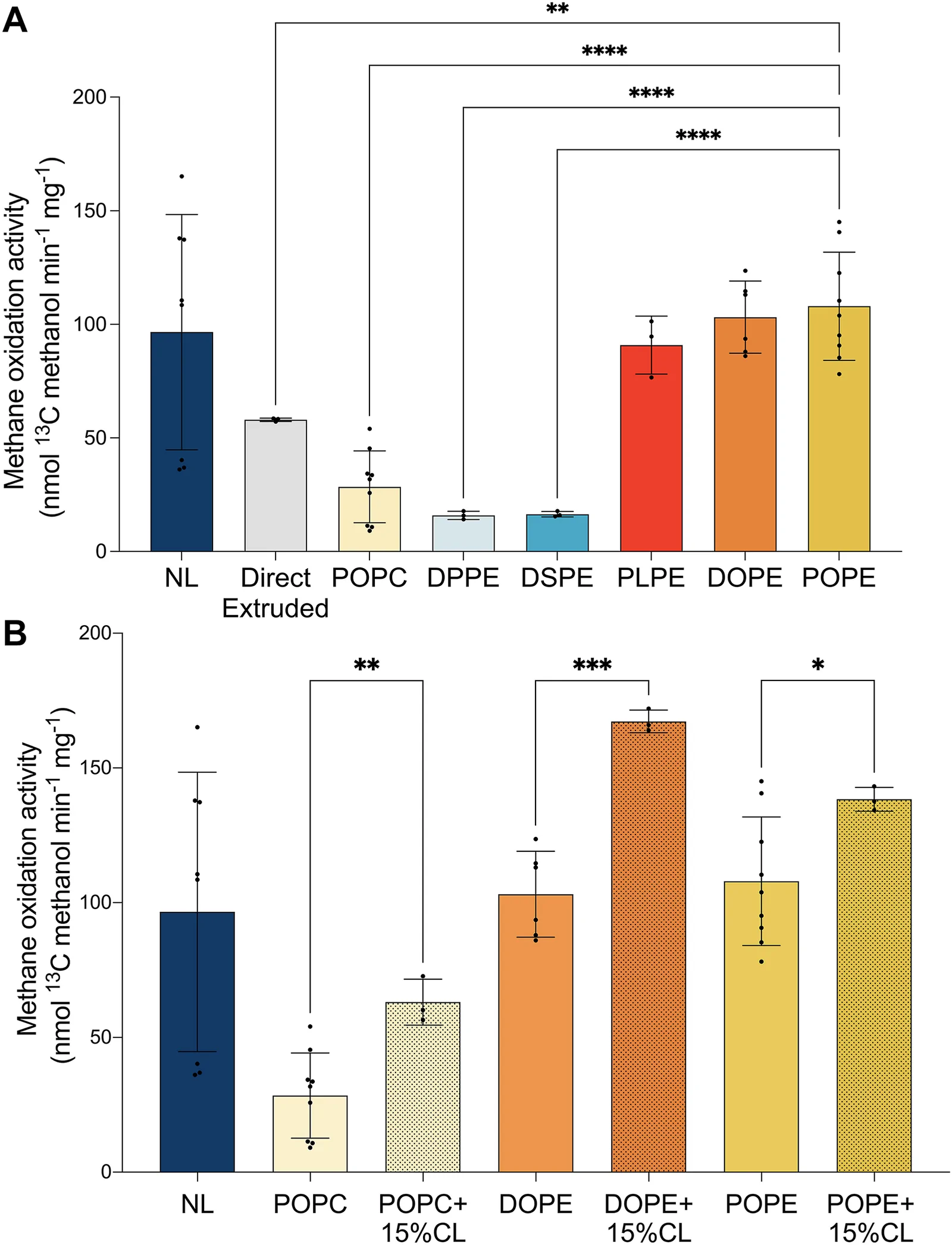
**Methane oxidation activity as a function of lipid composition.***A*, PE liposomes extruded at 50 nm. From *left to right*, technical replicates are as follows: n = 8, 3, 9, 3, 3, 3, 6, 9. *B*, POPC, DOPE, and POPE liposomes extruded at 50 nm with 15% cardiolipin (CL) doped in. From *left to right*, replicates are as follows: n = 8, 9, 3, 6, 3, 9, 3. Significant values are as follows, determined by one-way ANOVA: ∗ = *p* < 0.05, ∗∗ = *p* < 0.01, ∗∗∗ = *p* < 0.001, ∗∗∗∗ = *p* < 0.0001. Standard deviation and technical replicates also shown in Table S2.

To further investigate the correlation between PE lipids and activity, pMMO was reconstituted into liposomes containing PE lipids with different degrees of saturation and compared to reconstituted liposomes containing NL. NL-reconstituted 50 nm liposomes exhibited an average activity of 97 nmol ^13^CH_3_OH min^−1^ mg^−1^ (Fig. 5*A* and Table S2). Unsaturated lipids are more flared and conical in shape, resulting in a less tightly packed and more fluid membrane (39). In addition to PLPE, DOPE, and POPE, liposomes were prepared with 1,2-dipalmitoyl-sn-glycero-3-phosphoethanolamine (DPPE) and 1,2-distearoyl-sn-glycero-3-phosphoethanolamine (DSPE). DPPE and DSPE are cylindrical in shape due to their full saturation while POPE has one monounsaturated acyl chain, DOPE has two monounsaturated acyl chains, and PLPE has one diunsaturated acyl chain (Fig. 4). The unsaturation of PLPE, DOPE, and POPE lipids correlated with higher activity, with POPE yielding the highest activity of 108 ± 20 nmol ^13^CH_3_OH min^−1^ mg^−1^ in 50 nm liposomes, (Fig. 5*A* and Table S2). Notably, the polyunsaturation of DOPE and PLPE did not yield significant differences, indicating that little is gained by a second unsaturated bond. In liposomes extruded at 100 nm, the doubly monounsaturated DOPE lipids yielded the highest activity of any of the PE liposomes, 155 ± 48 nmol ^13^CH_3_OH min^−1^ mg^−1^ (Fig. S5 and Table S2).

Since *M. capsulatus* (Bath) cells also contain ∼14% CL (12, 14), the effects of doping 15% CL into POPC, POPE, and DOPE liposomes extruded at 50 nm were measured. CL addition approximately doubled the activity of pMMO in POPC liposomes from 29 ± 15 nmol ^13^CH_3_OH min^-1^ mg^-1^ for 100% POPC to 63 ± 7 nmol ^13^CH_3_OH min^-1^ mg^-1^ for 85% POPC + 15% CL liposomes (Fig. 5*B* and Table S2). For POPE, addition of 15% CL increased the activity from 108 ± 20 nmol ^13^CH_3_OH min^−1^ mg^−1^ to 138 ± 4 nmol ^13^CH_3_OH min^−1^ mg^−1^ with a decrease in activity upon increasing the CL to 25 or 50% (Fig. S6). Similarly, 100% DOPE liposomes extruded at 50 nm produced 103 ± 15 nmol ^13^CH_3_OH min^-1^ mg^-1^, which increased to 167 ± 3 nmol ^13^CH_3_OH min^−1^ mg^−1^ in the presence of 15% CL (Fig. 5*B* and Table S2). Like unsaturated lipids, CL is more conical in shape and localizes to curved membranes (40), consistent with the finding that increased surface curvature enhances pMMO activity.

**Figure 6:**
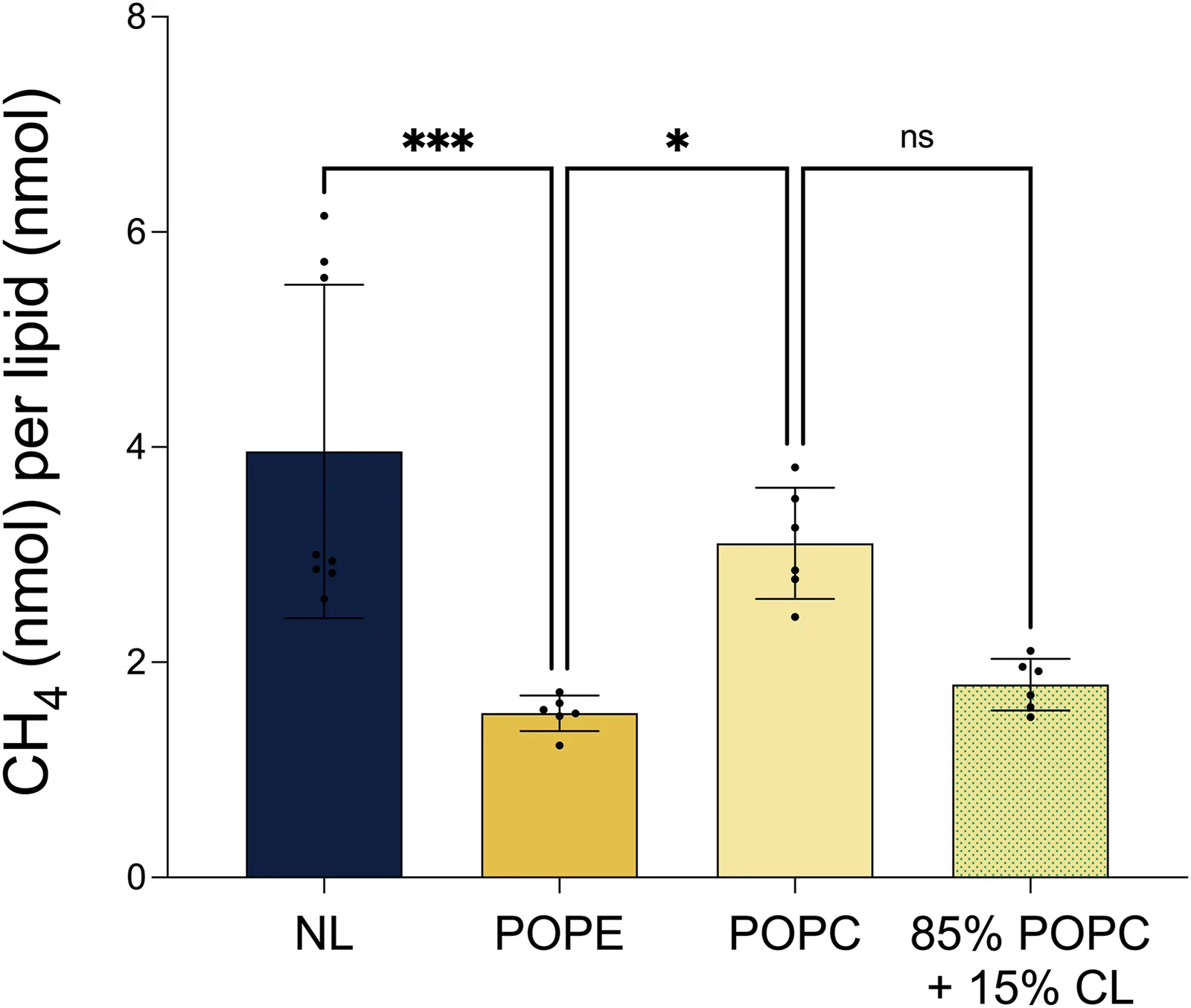
**Methane solubility as a function of liposome composition.** Solubilized methane (nmol) in 50 nm pMMO-reconstituted liposomes containing NL extracted from *M. capsulatus* (Bath) (n = 8), POPC (n = 6), POPC with 15% CL (n = 6), and POPE (n = 6). The amount of solubilized methane in nmol was normalized to lipid concentration in nmol. Significant values are as follows, determined by one-way ANOVA: ∗ = *p* < 0.05, ∗∗∗ = *p* < 0.001.

It is possible that the changes in membrane properties imparted by PE lipids, unsaturated acyl chains, and CL improve pMMO activity by increasing methane solubility. To test this, methane solubility was measured for 50 nm pMMO-reconstituted liposomes in the presence of the inhibitor acetylene (41, 42) to prevent methane removal by pMMO. The highest methane solubility is observed for NLs, with more methane per lipid than the POPC/CL and POPE compositions (Fig. 6). However, the difference in solubility between NL and POPC, between POPC and POPC/CL, and between POPC/CL and POPE is not significant. Moreover, the solubility of methane in POPE liposomes is less than in POPC liposomes (Fig. 6), in contrast to the increased activity with POPE (Figs. 3 and 5). While differences in solubility are modest, they are statistically significant as determined by one-way ANOVA analysis (Fig. 6). Together, these data indicate that while methane solubility varies in lipid bilayers of different compositions, there are other lipid-dependent factors that impact pMMO activity. Elucidating the molecular mechanism of these effects on activity is an important goal for future studies.

### pMMO interface in native membranes

One way that lipids could influence pMMO activity is by facilitating formation of the hexagonal arrays. To begin addressing this possibility, we used cryoEM to examine the interfaces between neighboring pMMO trimers in native membranes. While cryoET and cryoEM structures of a single *M. capsulatus* (Bath) pMMO trimer in the native membrane arrays have been determined to 4.8 Å (19) and 2.4 Å (12) resolutions, respectively, the surrounding trimers were not well resolved in the maps, precluding examination of trimer-trimer interactions. To obtain structural information on the interfaces, a cryoEM dataset was collected for native membranes from *M. capsulatus* (Bath). These data were initially processed according to previously described methods, focusing on gathering a stack of pMMO particles suitable for refinement (12). After obtaining a structure of pMMO in native membranes using this approach, the particles were examined using 2D classification in which particles from 2D classes were selected and centered on the interfaces between pMMO trimers. The best particles were used for ab initio refinement, creating a low-resolution map of the pMMO trimer interface, which was then used as a reference for heterogeneous refinement, gathering particles centered on the pMMO-pMMO interfaces. Several rounds of heterogeneous refinement and non-uniform refinement yielded a consensus map of the pMMO trimer interface (Fig. S7 and Table S3).

**Figure 7:**
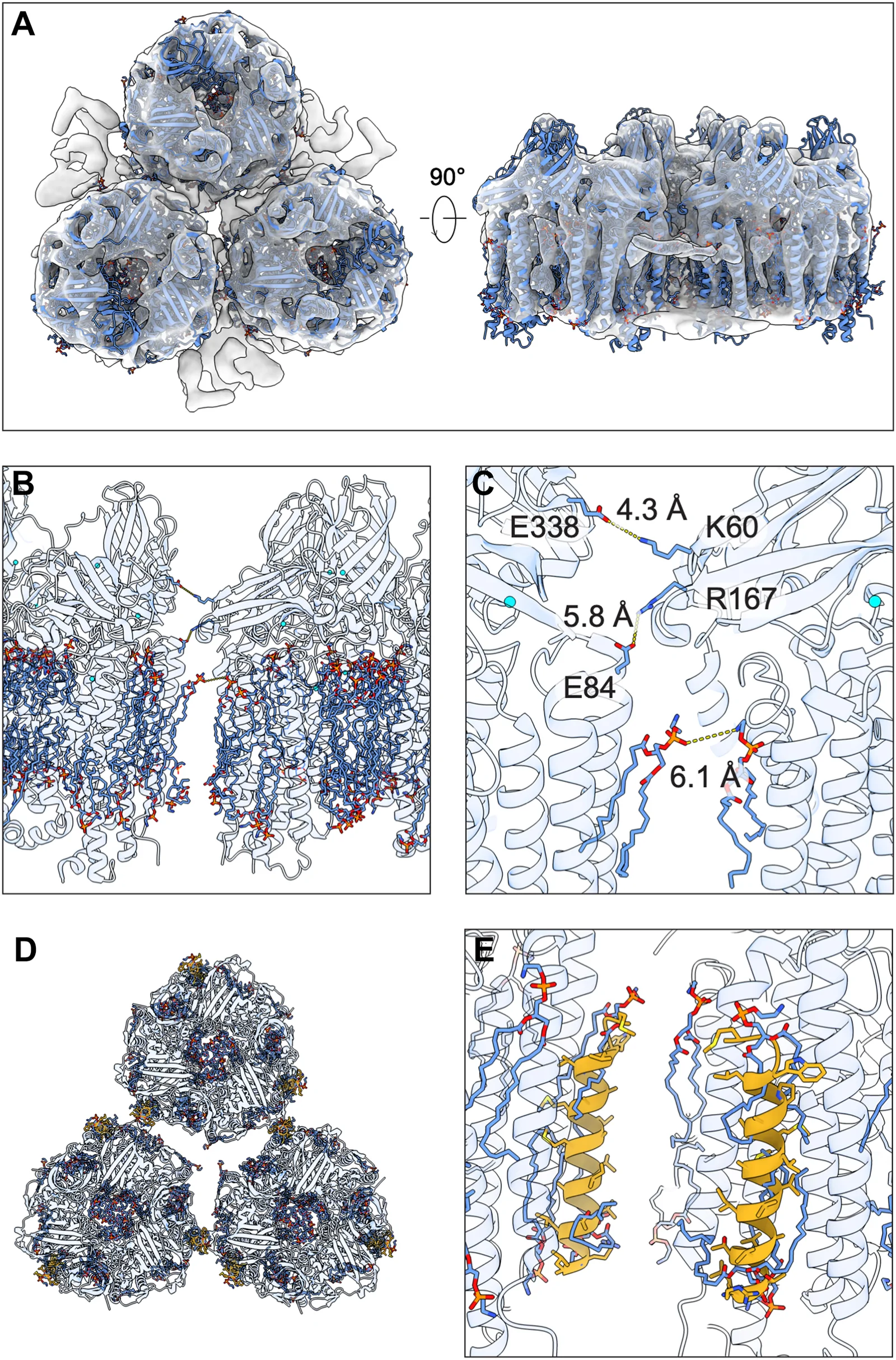
**CryoEM analysis of pMMO in native membrane arrays.***A*, pMMO models (PDB 9CL2) aligned in the C3 symmetrical cryoEM map with lipid molecules from the cryoEM structure shown. *B*, interface between two pMMO trimers showing closest possible contacts. *C*, zoomed-in view of interface in (*B*) showing the distances between pMMO models as fit into the cryoEM map. *D*, top-down view of PmoX helical subunit from *M.* sp. Rockwell superimposed on the *M. capsulatus* (Bath) trimer-of-trimers model. *E*, zoomed-in view of one interface.

Refinement of particles from 2D classes centered on pMMO interfaces using C1 symmetry leads to a single, off-center pMMO trimer resolved to high resolution (∼3.8 Å), with neighboring pMMO trimers appearing at lower resolution (Fig. S8). However, enforcement of C3 symmetry yields a trimer interface that shows each pMMO clearly (Fig. 7*A*) at ∼7.3 Å resolution, suggesting that the pMMO trimers are arranged in a C3 symmetrical or pseudo-symmetrical fashion as an asymmetrical arrangement of pMMO trimers would likely result in a blurred map with C3 symmetry imposed incorrectly. The moderate resolution of the interface maps compared to that of the single pMMO trimer map suggests that pMMO-pMMO interactions are not as ordered as the interprotomer interactions that mediate pMMO trimer formation. Instead, pMMO-pMMO interfaces may be mediated by weaker protein-protein interactions, protein-lipid interactions, or steric packing. It may also be that some pMMO-pMMO interfaces are asymmetrical, with randomly or non-uniformly arranged interfaces, but that these conformations are sorted or averaged away during cryoEM data processing.

Three copies of the *M. capsulatus* (Bath) pMMO trimer in native membranes (PDB 9CL2) were then modeled into this ∼7 Å C3 symmetry cryoEM map. In this trimer-of-trimers model, the PmoB Cu_B_ site is ∼40 Å from the nearest Cu_B_ of a neighboring pMMO, and ∼52 Å from the nearest Cu_D_ site in a neighboring pMMO, which is similar to the Cu_B_-Cu_D_ distance between protomers in a single trimer. These distances render the possibility of inter-pMMO electron transfer unlikely. The closest approach between copper sites is within a single protomer, with a Cu_B_-Cu_D_ separation of ∼22 Å.

Prior molecular dynamics simulations based on a 12 Å resolution cryoET trimer-of-trimers model suggested a strong salt bridge between PmoB residues Lys60 and Glu338 as well as more transient interactions between Lys58 and Glu160 and between Arg167 and Asp339 (19). While the 7 Å resolution map cannot resolve hydrogen bonds, possible side chain positions can be inferred from the location of the main chain in the current model and are consistent with the simulation results, with the closest rotamers of Lys60 and Glu338 separated by ∼4.3 Å and rotamers of the other two pairs within 8 Å of each other. In addition, PmoB residue Arg167 is within 6 Å of PmoC residue Glu84 on the adjacent trimer. This interaction, located at the interface between the periplasmic and transmembrane regions, neighbors a region of close interaction between PmoC subunits in which two lipid headgroups are within 6 Å of one another (Fig. 7, *B* and *C*). Notably, one side of this interface corresponds to the binding site for PmoX in the *M*. sp. Rockwell cryoEM structure (Fig. 7, *D* and *E*), supporting a role for supernumerary helices in mediating trimer interactions and potentially array formation. While not observed in this map, some higher-resolution cryo-EM maps of *M. capsulatus* (Bath) pMMO show a weak, unidentified helical density between protomers (12).

## Conclusion

These results set the stage for detailed molecular characterization of how the lipid environment facilitates copper membrane monooxygenase activity. The observation of higher pMMO activity in 50 nm liposomes compared to 100 nm liposomes suggests that decreased diameter, associated with increased curvature, enhances activity. Hexagonal patterning of pMMO arrays would be better accommodated by a planar surface, whereas a curved membrane might necessitate defects in the array pattern, yielding more truncated icosahedral (buckyball) shapes and perhaps facilitating access of substrate, reductant molecules, or partner proteins to pMMO. Both highly curved and planar membrane regions are observed by cryoET of *M. capsulatus* (Bath), *M.* sp. Rockwell, and *N. europaea* cells, but the relative distribution of pMMO/AMO in these regions is not clear and will require reliable particle picking of pMMO/AMO from tomograms, subtomogram averaging, and back-placement of pMMO/AMO particles into segmented tomograms, tasks which are limited by the relatively small size of pMMO/AMO for cryoET and the orientation bias imposed by the membrane.

The data also underscore the importance of lipid composition in the native membrane environment, with unsaturated PE lipids conferring increased activity, which may derive from their conical nature, consistent with the potential importance of membrane curvature. The proteolipid membrane can bend, compress and expand, all examples of curvature elastic deformation (22). Increased curvature elastic stress from non-lamellar forming lipids has a significant, often positive impact on membrane protein function (22, 26, 27, 43, 44, 45). The insertion of a membrane protein in these canonically non-lamellar-forming bilayers can relieve some of the curvature elastic stress, providing a more stable proteolipid bilayer than the lipid bilayer alone (26). Additionally, PE lipids can increase protein incorporation into liposomes (27). While the correlation between lipid identity and activity is clear, it remains a future challenge to disentangle the relative contributions of lipid headgroups, acyl chain saturation, and lipid shape to enzyme activity. Notably, addition of CL further enhances activity. Whether CL interacts directly with pMMO or modulates the membrane bilayer structure remains to be determined.

Lipids are closely associated with pMMO and AMO in native membranes (12), and the cryoEM trimer-of-trimers model determined here reveals close interactions between lipids associated with neighboring pMMOs. Thus, the lipid identity may modulate enzyme conformation or accessibility to the active site. CryoEM characterization of pMMO in liposomes of defined composition will help address these questions in the future. Overall, understanding lipid-pMMO interactions necessary for methane oxidation in methanotrophs will guide the rational design of pMMO-based strategies for methane capture and remediation. More broadly, this work highlights the notion that the membrane environment is not a passive structure, but a key contributor to the enzymatic activity.

## Experimental procedures

### Bacterial growth

*M. capsulatus* (Bath) and *M*. sp. Rockwell were grown at 45 °C and 30 °C, respectively, in a 12 L fermentor (BioFlo 4500, New Brunswick Scientific). Media components contained 1x nitrate mineral salts, 3.9 μM phosphate buffer, pH 6.8, 40 μM NaFe(III)EDTA, 50 μM CuSO_4_, and trace elements, including Mo, Zn, Mn, Co, Ni and H_3_BO_3_. Cells were grown with a 1:3 methane:air flow ratio at 1 L/min and agitated at 200 rpm. After reaching an OD_600_ of 8 to 10, cells were harvested via centrifugation at 5000*g* for 30 min, and cell paste was stored at −80 °C. *N. europaea* ATCC 19718 was grown and harvested as described previously (12).

### Isolation of membranes and pMMO solubilization

Frozen *M. capsulatus* (Bath) cell paste (20 g) was resuspended in a 25 mM PIPES, 250 mM NaCl, pH 7.4 (lysis buffer), and cells were lysed via sonication (QSonica Q700) for 10 min using a power amplitude of 45, with 1 s on and 1 s off. The lysed cells were centrifuged at 10,000*g* for 45 min to remove cell debris. The membranes were then isolated *via* ultracentrifugation at 150,000*g* for 1 h. In three subsequent washing steps, the membranes were resuspended in lysis buffer in a Dounce homogenizer and ultracentrifuged again at 150,000×*g* for 30 min. The final membrane pellet was resuspended in 7 ml of lysis buffer, aliquoted, frozen in liquid nitrogen and stored at −80 °C until use.

Detergent-solubilized pMMO was extracted from *M. capsulatus* (Bath) membranes by adding n-dodecyl-β-D-maltopyranoside (DDM) to the isolated crude membranes in a 1.2 mg DDM to 1 mg pMMO ratio, followed by gentle mixing (15 rpm) at 4 °C for 1 h. To remove any precipitate or remaining membranes, this mixture was then centrifuged at 150,000*g* for 1 h at 4 °C. The supernatant containing the solubilized pMMO was collected and buffer exchanged (Amicon, 100 kDa) into 25 mM PIPES, 250 mM NaCl, pH 7.4 buffer containing 0.02% DDM. The solubilized pMMO was then aliquoted, frozen in liquid nitrogen, and stored at −80 °C until use. The concentration of protein in both the membranes and the solubilized pMMO was determined using the DC Lowry assay (Bio-Rad) with BSA as the standard.

### Liposome preparation and analysis

Liposomes were prepared by two different methods. For direct extrusion of liposomes from native membranes, 0.3 mg/ml crude membranes in 25 mM PIPES, 250 mM NaCl, pH 7.4 were extruded using an Avanti Polar Lipids Mini Extruder. Two polycarbonate filter papers were used (400, 100, and 50 nm pore sizes), and the membranes were passed through the extruder 11 times. For the second method, synthetic lipid solutions and native lipid extracts were prepared as described previously (14). Liposomes formed with synthetic lipid mixtures or native methanotroph lipids (but lacking protein) were extruded by 11 passes through two 100 and 50 nm filters on the Avanti Mini Extruder. These liposomes were then destabilized with 0.04% DDM for 1 h at room temperature. Detergent-solubilized pMMO was then added in a 1:5 M ratio (protein:lipid) and mixed for 1 h at 4 °C. Pre-wetted (PIPES/NaCl buffer) BioBeads were added (0.8 g/1 ml sample) and mixed for 60 to 90 min. The sample was decanted from the BioBeads and buffer exchanged in PIPES/NaCl. Liposomes were used immediately and not frozen. DLS was performed with a pUNK instrument from Unchained Labs using a BladeCell in which 5 μl of sample was measured for 10 s with 10 repeats per sample.

Protease digestion of the liposomes was performed on reconstituted liposomes with solubilized pMMO and native lipids (NL) extracted from *M. capsulatus* (Bath). Both NL liposomes (lipo, Fig. S3) and isolated intracytoplasmic membranes (crude, Fig. S3) at approx. 2 μM protein concentration were treated with 8.5 μM of trypsin in PIPES/NaCl buffer. The reaction was performed at room temperature for 2 h before loading onto an SDS-PAGE gel for analysis.

Proteomic analysis was performed on the reconstituted liposomes with solubilized pMMO from *M. capsulatus* (Bath) and synthetic POPE lipids. 200 μl of 1 μM liposomes in PIPES/NaCl buffer were submitted to the Proteomics Core at Northwestern University. Samples were analyzed with DDA Scaffold.

Lipid to pMMO ratios were determined for 10 compositions of 50 nm reconstituted liposomes (Fig. S4). Each liposome solution was prepared as reported above, and then pelleted *via* ultracentrifugation for 30 min at 115,000*g*. The pelleted liposomes were gently resuspended in 200 μl of PIPES/NaCl buffer. The protein and lipid contents of these liposomes were measured by the DC Lowry assay (BioRad) and a fluorescent lipid quantification kit (Cell BioLabs, STA617), respectively. Molar concentrations were compared and reported in Fig. S4.

### GC-MS methane oxidation activity assay

Activity assays monitoring conversion of ^13^CH_4_ to ^13^CH_3_OH were performed using approximately 0.2 mg/ml of pMMO in liposomes. Each assay, performed in triplicate, was accompanied by controls that included triplicate samples with no enzyme, triplicate samples with no NADH, and triplicate samples with no ^13^CH_4_ to ensure that detected ^13^CH_3_OH derived from pMMO activity. For each assay, 98 μl of sample was loaded into a gas-tight GC-MS vial with 2 μl of 200 mg/ml NADH. 1 ml of airspace was replaced with 1 ml of ^13^CH_4_ and allowed to react for 5 min at 45 °C. Reactions were quenched with 1 mM dichloromethane (DCM) in chloroform, and DCM was used as an internal standard in data analysis. After quenching, samples were mixed for 10 min at 2000 rpm and centrifuged for 1 h at 2000*g*. An Agilent 7890B/5977 A MSD GC/MS instrument with a PoraBOND Q column (25 m × 250 μm × 3 μm) was used for ^13^CH_3_OH analysis against a standard curve, as described previously (12, 14).

### GC-FID methane solubility measurements

Liposome suspensions were placed into gas-tight vials with butyl stoppers. 1% acetylene and 10% methane were injected in the headspace v/v in a Balch tube. After incubation for 24 h at room temperature with 200 rpm shaking, 200 μl of the saturated liposome suspension was transferred into a new sealed vial (37 ml), vortexed, and incubated for 2 h for headspace equilibration. 1 ml of the headspace from each vial was injected into an SRI 8610C GC (SRI Instruments) equipped with a 6′ silica gel column at an oven temperature of 80 °C and a flame ionization detector at 385 °C. The carrier gas consisted of >99.998% N_2_ gas (Praxair) at 20 ml/min, >99.5% H_2_ gas (Praxair) at 25 ml/min, and air supplied *via* an internal pump at 250 ml/min. Methane solubility was normalized to lipid concentration using the Cell BioLabs fluorescence lipid quantification kit (STA617). Data were collected twice in triplicate.

### CryoFIB milling and cryoET

CryoFIB milling and cryoET were performed at the Midwest Center for Cryo Electron Tomography (MCCET). Cells were diluted to an OD_600_ of 2 with 25 mM PIPES, 250 mM NaCl, pH 7.4, and 3 μl were applied to glow-discharged Quantifoil 300 mesh Cu R2/1 grids. The samples were incubated at 100% humidity in 4 °C for 30 s and then dual-side blotted (Vitrobot) for 4 s before plunging into liquid ethane for freezing. Grids were stored under liquid nitrogen until use. Clipped grids were loaded into a TFS Aquilos 2 Cryo FIB using the AutoTEM Cryo 2.4 software. Platinum sputtering (20 mA, 25 s, 30–50 nm) and organometallic platinum coating (3 μm) was performed. Milling sites were set up with a targeted lamella thickness of 200 nm (Table S4). Milled grids were stored under liquid nitrogen until data collection on a TFS Titan Krios G4 operated at 300 kV with a Falcon 4i Direct electron detector using SerialEM for data collection (Table S5).

### CryoET data processing and segmentation

CryoET data was processed through EMAN2 following the published workflow (46, 47). Briefly, a neural network was trained for each dataset by manually segmenting ∼30 training references within each tomogram. The neural network was then applied to each tomogram to produce the segmentations. The tomograms and reconstructed segmentations were visualized in ChimeraX (48).

### Negative stain EM

Negative stain images were collected on a JEOL1400 microscope at 30k magnification with a 3.71 Å pixel size. 400-mesh Cu grids were glow-discharged and prepared via uranyl acetate staining. Membranes at 0.5 mg/ml (3 μl) were applied to the grid and subsequently washed in four droplets of 1% uranyl acetate. Grids were manually blotted and allowed to dry.

### CryoEM of pMMO in native membranes

Native membranes from *M. capsulatus* (Bath) were isolated and applied to cryoEM grids as reported previously (12). Isolated membranes were applied to cryoEM grids at 4 to 5 mg/ml using C-Flat holey carbon copper grids with 1.2 μm holes, 1.3 μm spacing, and 400 mesh (Electron Microscopy Sciences) with an FEI Vitrobot. The grids were first plasma cleaned using a Solarus plasma cleaner (Gatan) for 10 s at a voltage of 10 W. 3 μl of sample were applied to the grids under 100% humidity at 4 °C. Grids were then blotted for 4 s with a wait time of 30 s and vitrified in liquid ethane. CryoEM data were acquired at the National Center for CryoEM Access and Training (NCCAT) using a Titan Krios microscope operated by Leginon software. Detailed information about the cryoEM data collection and analysis parameters is provided in Table S3.

### CryoEM data processing of pMMO trimer interfaces

To gather a collection of pMMO particles suitable for 3D refinement focusing on pMMO-pMMO interfaces, the cryoEM structure of pMMO in native membranes was first determined using cryoSPARC (49) and previously published methods (12). Extensive 2D classification was performed to obtain classes that were centered on pMMO-pMMO interfaces. The best of these classes were then used for ab initio reconstruction to obtain a preliminary, low-resolution map of the pMMO-pMMO interfaces. This map was used as a reference for heterogeneous refinement to gather more particles. A previously published map of pMMO in native membranes (EM Map EMD-45659) was then shifted in ChimeraX to align with the low-resolution pMMO trimer interface map. This shifted map was used as a reference for further heterogeneous and non-uniform refinements in cryoSPARC to gain a higher quality stack of pMMO trimer interface particles. These refinements were performed with both C1 and C3 symmetry enforcements. The resulting particles were then re-extracted and recentered prior to additional refinement steps, including global and local CTF refinements. The final model (PDB 9CL2) was placed in the map for visualization.

## Data availability

Data will be made available upon publication; models have been deposited to the Protein Data Bank (PDB ID: 11LL) and the corresponding cryoEM maps have been deposited to the Electron Microscopy Data Bank (EMD: 75807). All other data are available within the main text, supporting information, or upon request.

## Supporting information

This article contains supporting information.

## Conflict of interest

The authors declare that they have no conflicts of interest with the contents of this article.

## Supporting information

Supporting File 1

Liposome_paper_SI
