# Supplementary material for: Membrane properties modulate methane oxidation by particulate methane monooxygenase
Source: J Biol Chem. 2026 Aug 12;302(10):113435. doi: 10.1016/j.jbc.2026.113435 (PMC13578624; doi:10.1016/j.jbc.2026.113435)
Supplement: Supplementary file 1 [file mmc2.pdf]

Supporting Information

Membrane properties modulate methane oxidation by particulate methane monooxygenase

Callie G. Miller[1], Frank J. Tucci[1], Genevieve R. Nemeth[1], Sergey Stolyar[2], Mary E. Lidstrom[2], and Amy C. Rosenzweig[1,*]

[1]*Departments of Molecular Biosciences and of Chemistry, Northwestern University, Evanston, IL, 60208, USA* [2]*Departments of Chemical Engineering and of Microbiology, University of Washington, Seattle, WA, 98195, USA*

*To whom correspondence should be addressed. E-mail: amyr@northwestern.edu

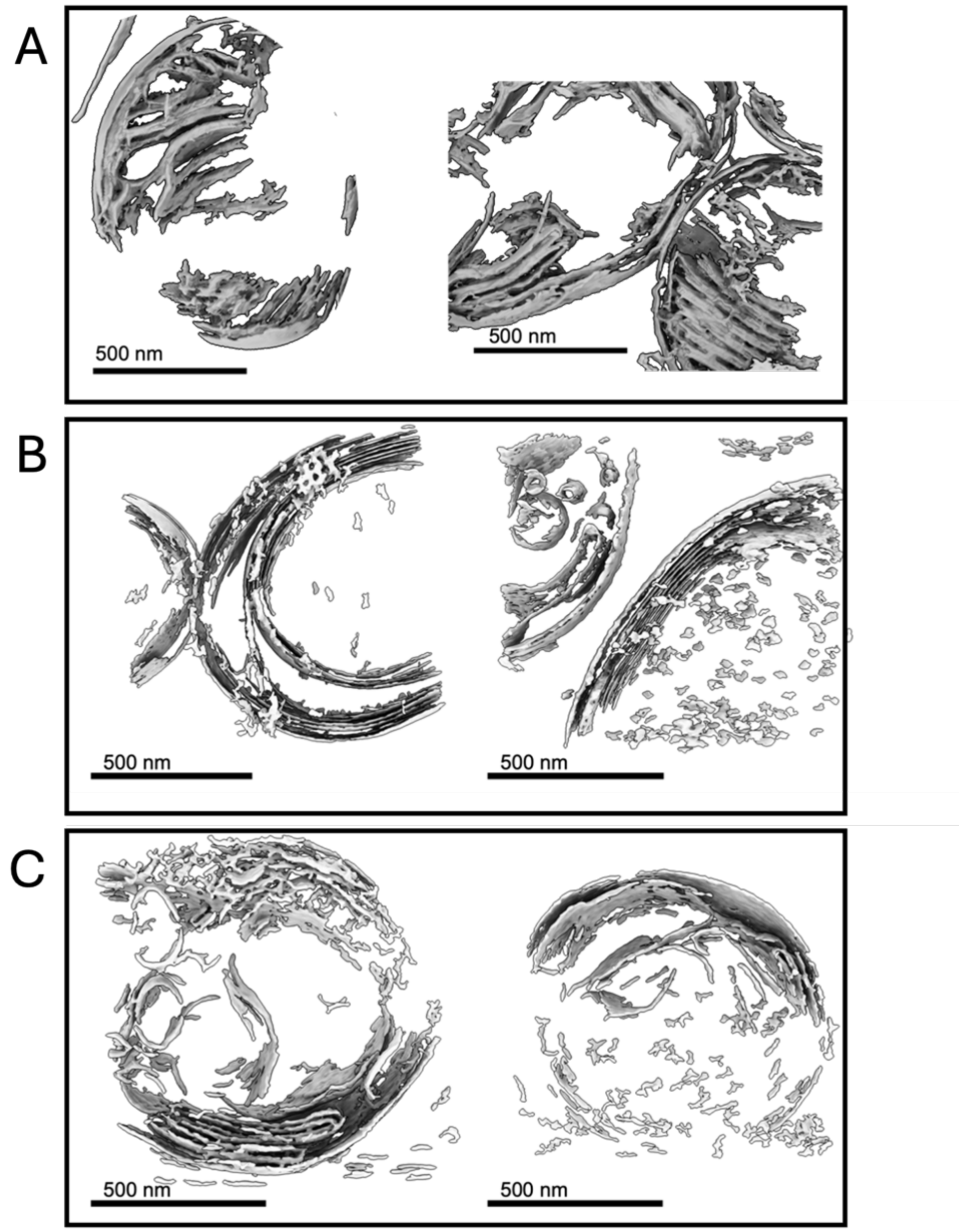

Figure S1. Alternative cryoFIB milled cells visualized by cryoET. *A*, *M. capsulatus* (Bath) cells. *B*, *M.* sp. Rockwell cells. *C*, *N. europaea* cells.

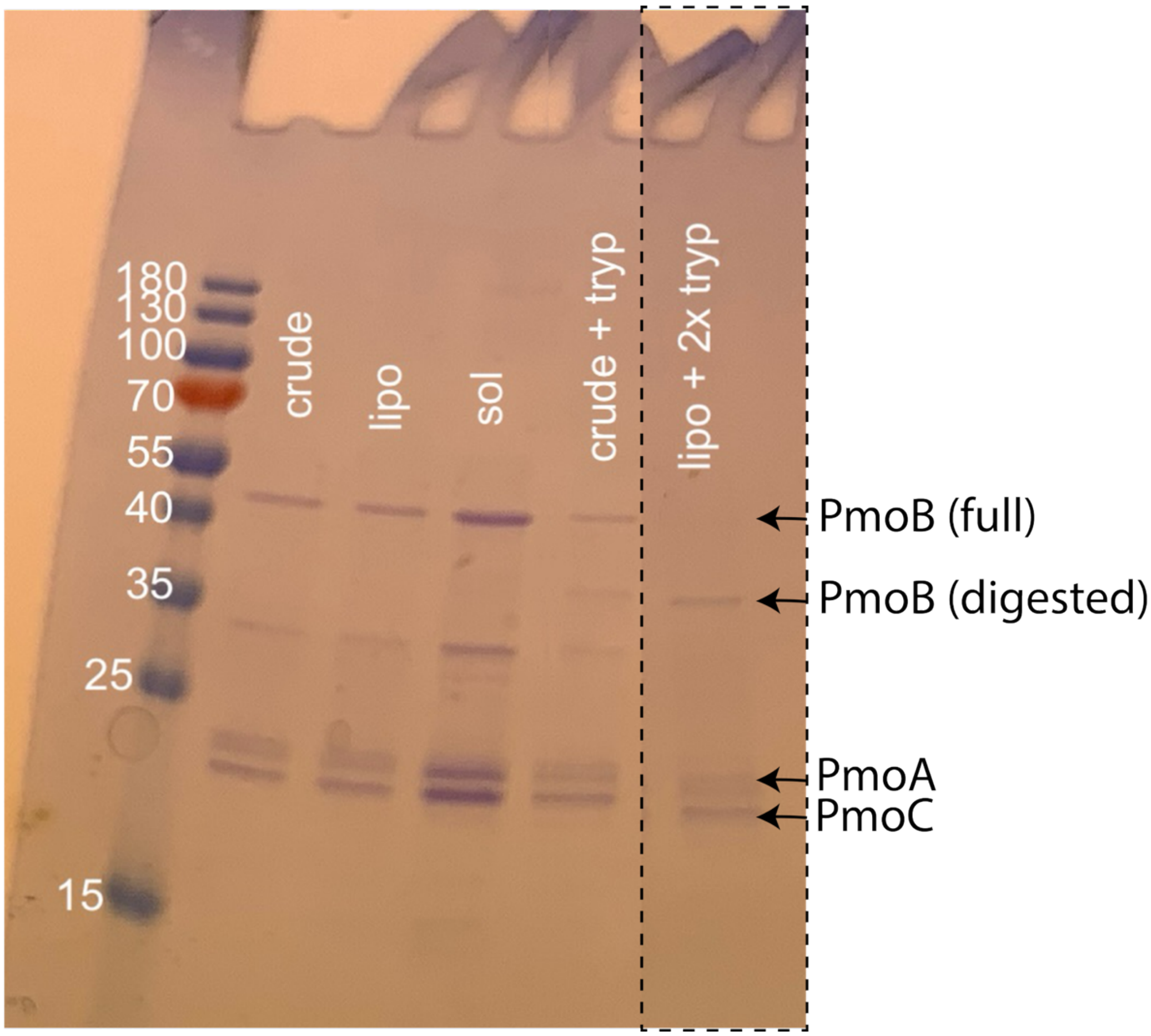

Figure S2. SDS-PAGE gel of isolated membranes and liposomes. Molecular weights are shown in kDa on the left ladder. Samples include crude, isolated intracytoplasmic membranes from *M. capsulatus* (Bath); lipo, reconstituted liposomes with pMMO and native lipids extracted from *M. capsulatus* (Bath); sol, solubilized pMMO used for liposome reconstitution from *M. capsulatus* (Bath); tryp, samples treated with 8.5 μM trypsin. Splice mark from the gel shown in the dashed black line, where relevant lanes from the same gel were spliced together.

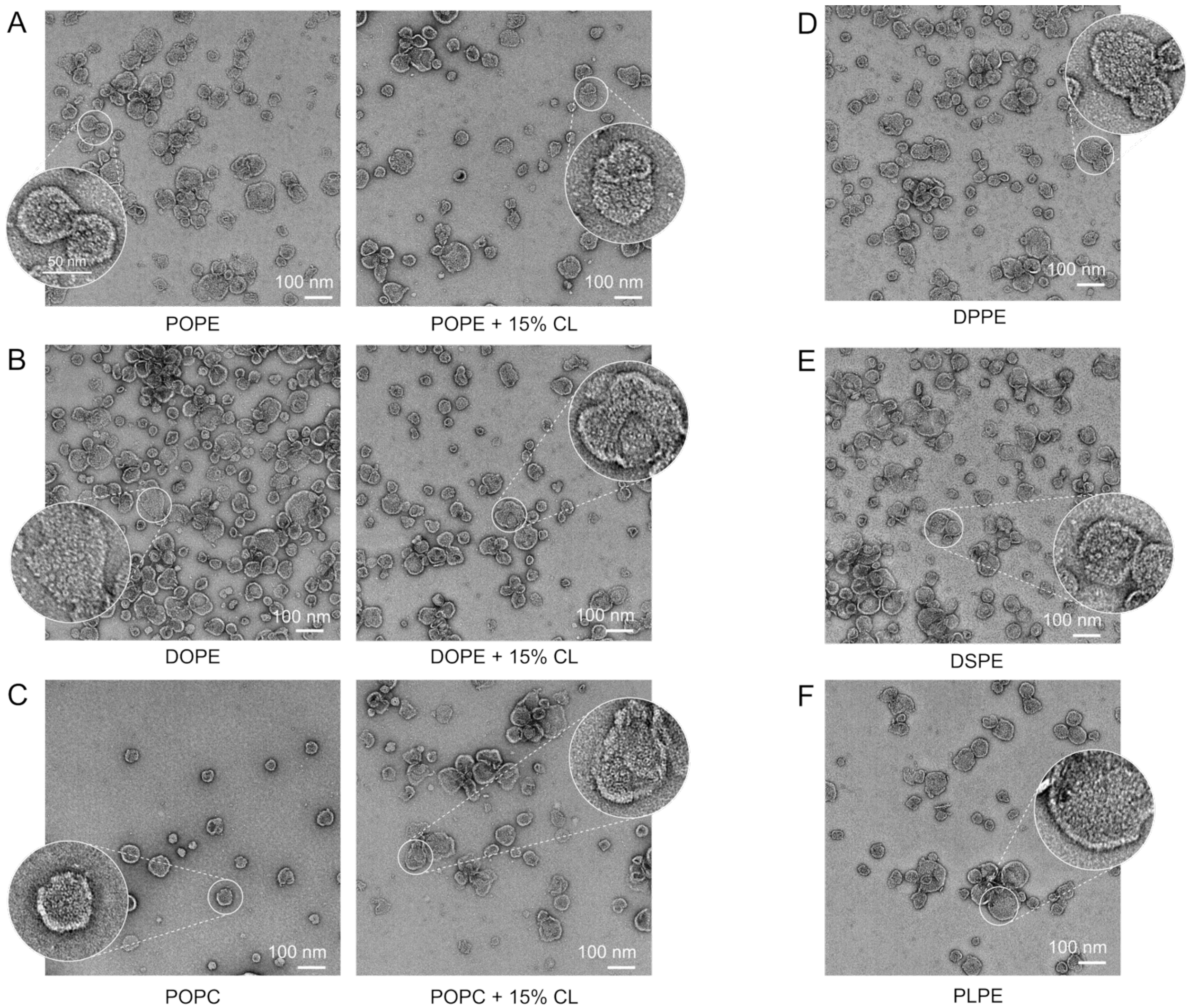

Figure S3. Negative stain micrographs of reconstituted liposomes. Solubilized pMMO was reconstituted into 50 nm extruded liposomes composed of synthetic lipids, including *A,* POPE (left) and POPE with 15% CL (right), *B,* DOPE (left) and DOPE with 15% CL (right), *C,* POPC (left) and POPC with 15% CL (right), *D,* DPPE, *E,* DSPE, and *F,* PLPE. Intact liposomes and pMMO arrays visible in each composition, where the circle highlights point out clear examples of the pMMO clusters.

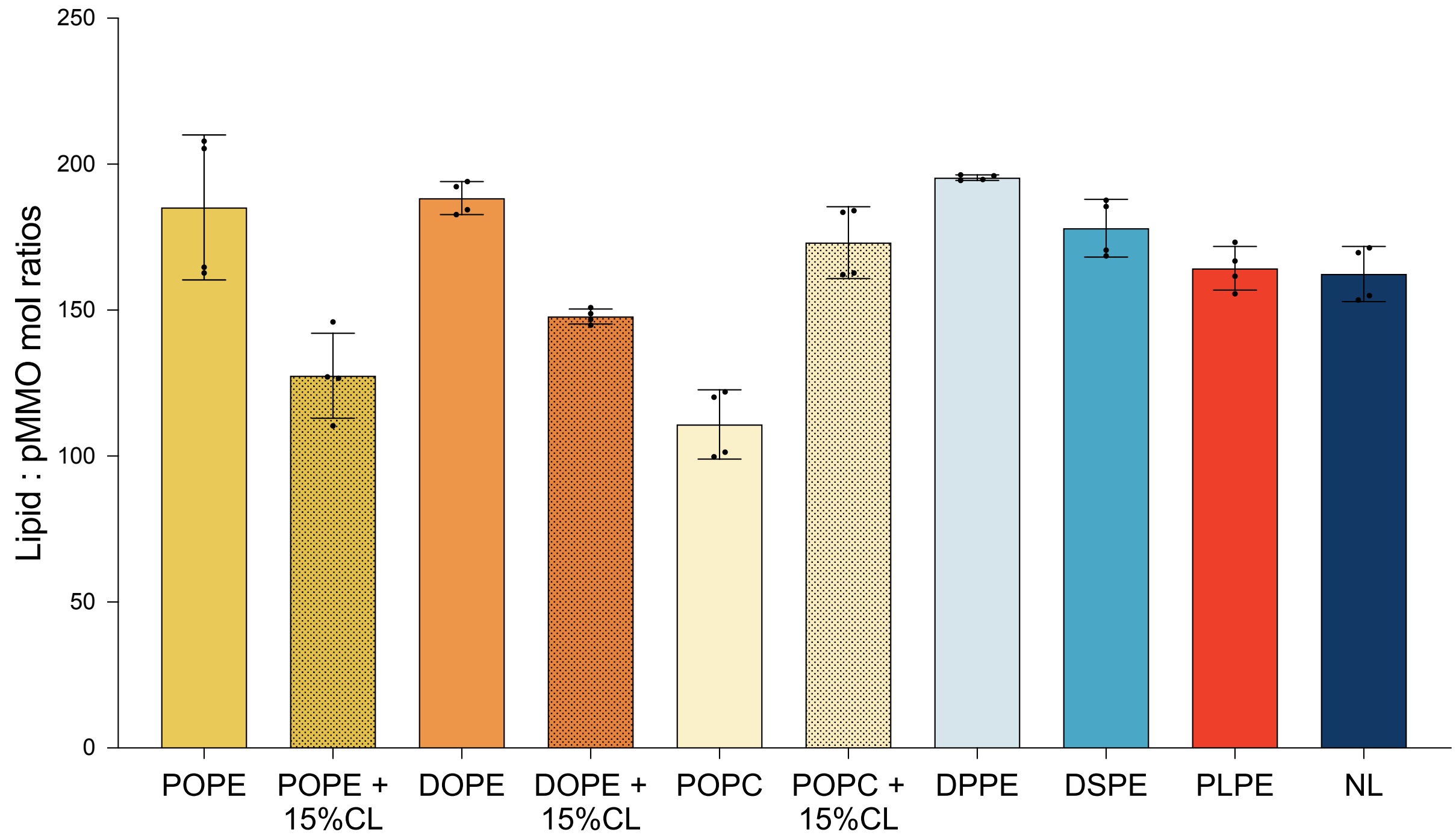

Figure S4. Lipid to pMMO ratios for liposomes of varying composition. Lipid to pMMO molar ratios are shown comparing 50 nm liposomes for different lipid compositions (n = 4), where pMMO concentration was determined in duplicate by a DC Lowry assay and lipid concentrations were determined in duplicate by a Cell BioLabs lipid quantification kit.

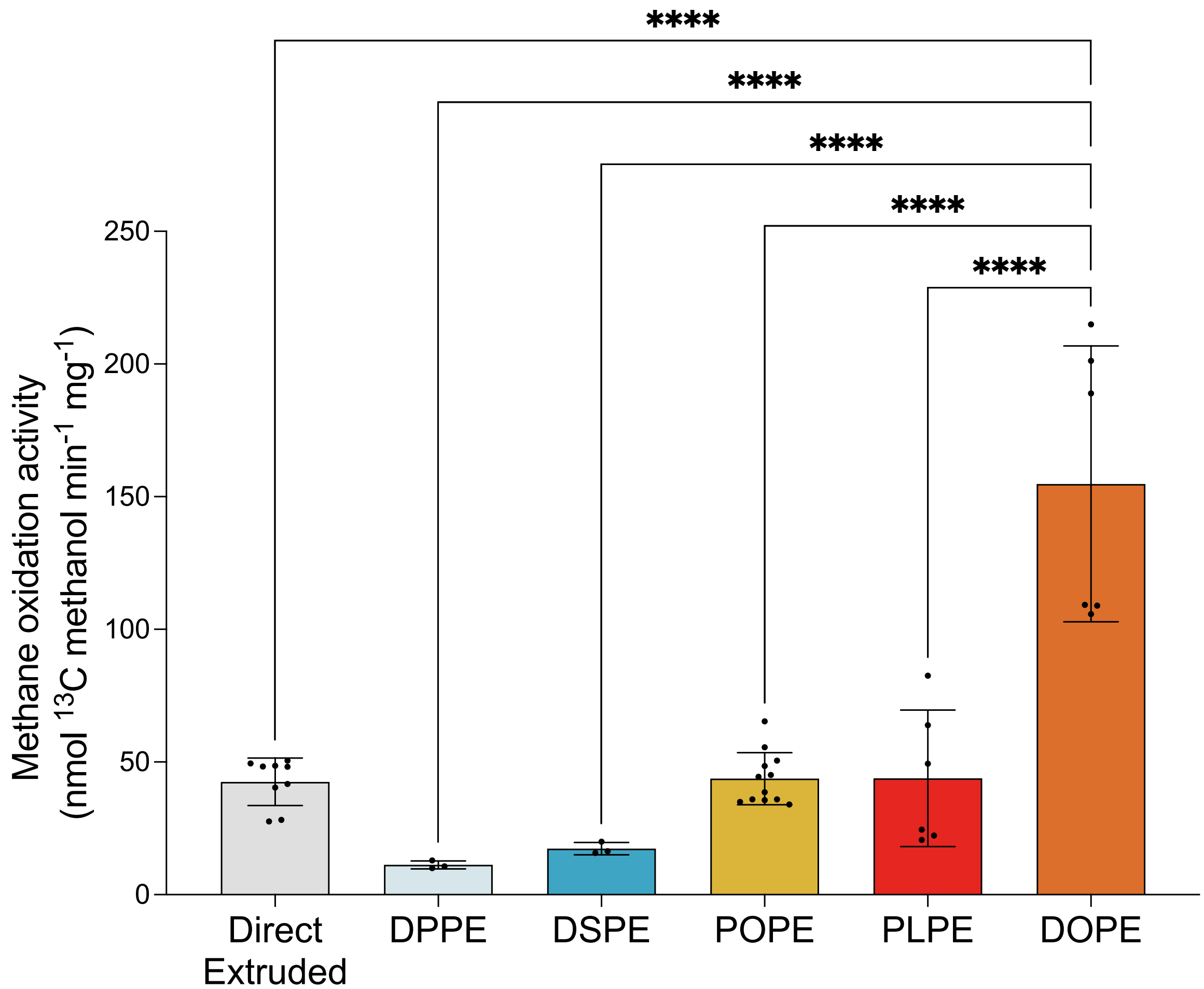

Figure S5. Methane oxidation activity as a function of lipid composition. PE liposomes were extruded at 100 nm. From left to right, n = 9, 3, 3, 12, 6, and 6. Significant values are as follows, determined by one-way ANOVA: **** = $p < 0.0001$.

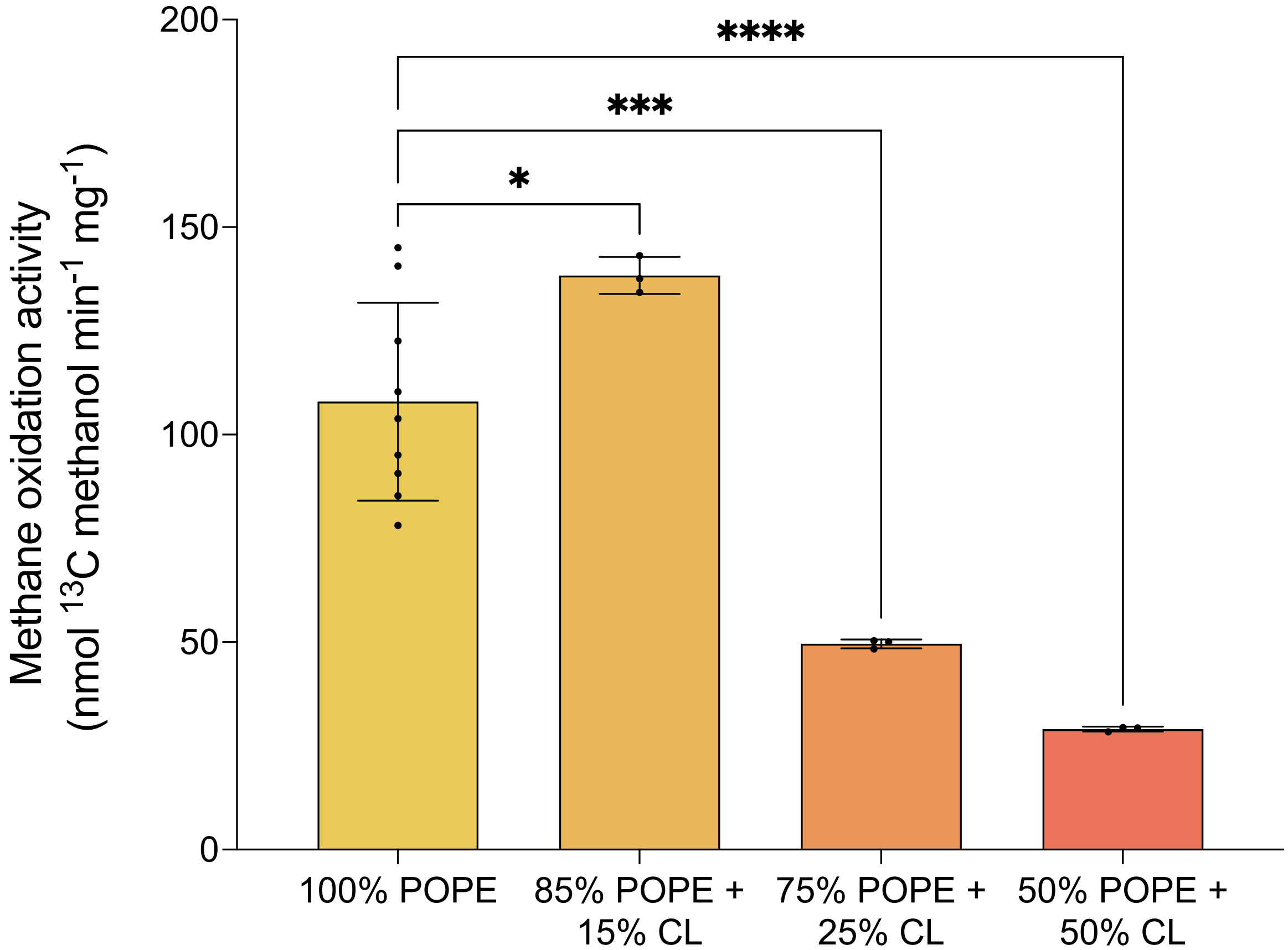

Figure S6. Methane oxidation activity with increasing cardiolipin. POPE reconstituted liposomes with increasing cardiolipin content were extruded at 50 nm. From left to right replicates n = 9, 3, 3, and 3. Significant values are as follows, determined by one-way ANOVA: * = $p < 0.05$, *** = $p < 0.001$, **** = $p < 0.0001$.

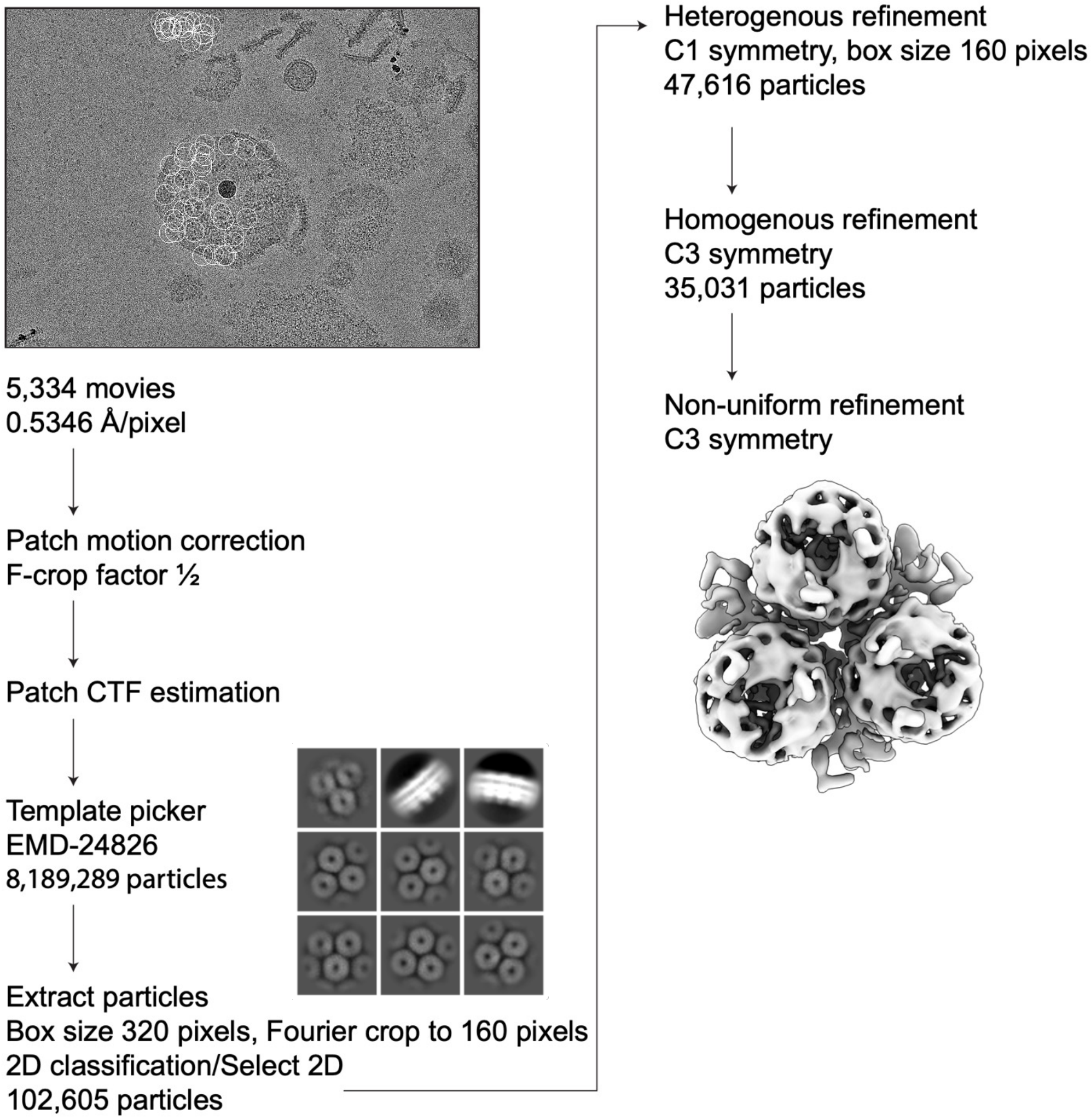

Figure S7. CryoEM data processing flowchart for pMMO arrays.

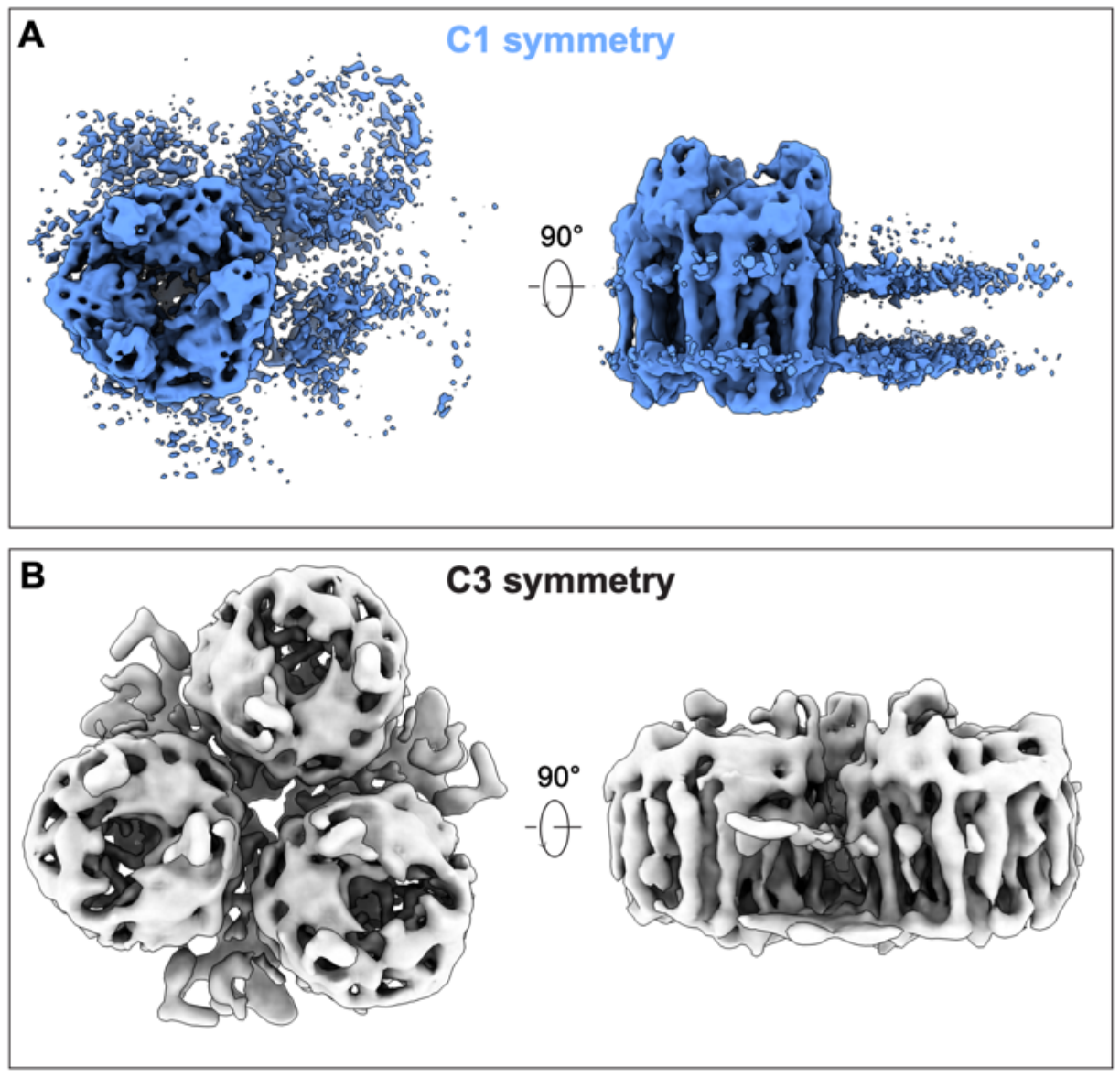

Figure S8. CryoEM maps of pMMO trimer interfaces in native membranes. *A*, C1 symmetry applied. *B*, C3 symmetry applied.

Table S1. Liposome diameters determined by DLS, average of 10 x 10 s measurements

Identity	Diameter (nm)	Std. Dev (nm)
Unextruded	276	142
400 nm direct extruded	231	114
100 nm direct extruded	127	48
50 nm direct extruded	100	4
50 nm POPC	112	53
50 nm POPC + 15%CL	124	30
50 nm POPE	98	45
50 nm DOPE	115	45
50 nm PLPE	122	65
50 nm NL	82	33
100 nm DPPE	132	70
100 nm DSPE	132	70
100 nm POPE	143	72
100 nm DOPE	192	45
100 nm PLPE	162	171
100 nm POPC	132	83
100 nm NL	114	46

Table S2. Methane oxidation values for various liposome compositions and sizes

Identity	Average activity (nmol ^{13}C methanol mg^{-1} min^{-1})
Unextruded	70 ± 45 (n = 15)
400 nm direct extruded	40 ± 2 (n = 3)
100 nm direct extruded	43 ± 8 (n = 9)
50 nm direct extruded	58 ± 1 (n = 3)
50 nm POPC	29 ± 15 (n = 9)
50 nm POPC + 15% CL	63 ± 7 (n = 3)
50 nm DOPE	103 ± 15 (n = 6)
50 nm DOPE + 15% CL	167 ± 3 (n = 3)
50 nm DPPE	15 ± 1 (n = 3)
50 nm DSPE	16 ± 1 (n = 3)
50 nm POPE	108 ± 20 (n = 9)
50 nm POPE + 15% CL	138 ± 4 (n = 3)
50 nm POPE + 25% CL	50 ± 1 (n = 3)
50 nm POPE + 50% CL	29 ± 1 (n = 3)
50 nm PLPE	91 ± 10 (n = 3)
50 nm NL	97 ± 49 (n = 8)
100 nm DPPE	11 ± 1 (n = 3)
100 nm DSPE	17 ± 2 (n = 3)
100 nm POPE	44 ± 9 (n = 12)
100 nm PLPE	44 ± 24 (n = 6)
100 nm DOPE	155 ± 48 (n = 6)
100 nm POPC	30 ± 8 (n = 3)
100 nm NL	21 ± 2 (n = 6)

Table S3. CryoEM data collection parameters and map and model validation statistics

Data collection and processing	
Movies (no.)	5,334
Magnification	105,000x
Voltage (kv)	300
Electron exposure (e^{-}/Å^2)	65.71
Defocus range (µm)	-0.8 to -2.5 µm
Pixel size (Å)	0.5346
Symmetry imposed	C3
Initial particle images (no.)	8,189,289
Final particle images (no.)	35,031
Map resolution (Å)<br>Fsc threshold 0.143	7.3
Map resolution range (Å)	6.455, 11.305, 14.300, 15.608, 23.618 (min, 25th percentile, median, 75th percentile, max)
Energy filter slit width	20 eV
**Refinement**	
Initial model used (pdb code)	Built de novo
Model resolution (Å)<br>Fsc threshold 0.143	7.16
Model resolution range (Å)	6.06-12.97
Model composition<br>Non-hydrogen atoms<br>Protein residues	 62874<br>7758
B factors (Å^2)	30.00
R.m.s. Deviations<br>Bond lengths (Å)<br>Bond angles (°)	 0.006 (0)<br>1.151 (44)
**Validation**	
Molprobity score<br>Clashscore<br>Poor rotamers (%)	2.21<br>7.73<br>4.12
Ramachandran plot<br>Favored (%)<br>Allowed (%)<br>Outliers (%)	 95.34<br>4.31<br>0.35

Table S4. CryoFIB milling parameters

Microscope	TFS Aquilos 2 Cryo FIB						
Targeted lamella thickness	200 nm						
Software	AutoTEM Cryo 2.4						
	Milling					Thinning	
	Stress relief cuts	Rough milling	Medium milling	Fine milling	Finer milling	Polishing 1	Polishing 2
Trench width	0.3 µm	-	-	-	-	-	-
Trench height	5 µm	-	-	-	-	-	-
Trench offset	3.5µm	-	-	-	-	-	-
Depth correction	60%	50%	160%	160%	160%	100%	75%
Milling current	0.1 nA	0.3 nA	0.1 nA	50 pA	50 pA	10 pA	10 pA
Milling pattern	-	Rectangular	CleaningCrossSection	CleaningCross Section	CleaningCross Section	CleaningCrossSection	CleaningCrossSection
Pattern overlap	-	-	800%	400%	200%	200%	200%
Pattern offset (µm)	-	1.5	0.8	0.3	0.25	0.05	0
Front width overlap (µm)	-	1.5, 1.5	0.65,0.65	0.35,0.35	0.05,0.05	-	-
Rare width overlap (nm)	-	1.1	0.5,0.5	0.1,0.1	0.05,0.05	-	-

Table S5. TEM data collection parameters

**Sample**	***M. capsulatus* (Bath)**	***N. europaea***	***M.* sp Rockwell**
**Microscope**	TFS Titan Krios G4		
Software	SerialEM	SerialEM	SerialEM
Voltage (kV)	300	300	300
Spherical aberration (mm)	2.7	2.7	2.7
Detector	Falcon 4i Direct electron detector	Falcon 4i Direct electron detector	Falcon 4i Direct electron detector
Energy filter, slit width	TFS Selectris, 10ev	TFS Selectris, 10ev	TFS Selectris, 10ev
Detector mode	Counting mode, EER	Counting mode, EER	Counting mode, EER
Frames per tilt image	1908	333	432
Magnification	19,500	53,000	53,000
Pixel size (data collected) (Å)	6.285	2.394	2.394
Defocus (µm)	-6	-5 to -6	-3.5 to -5.5
Tilt series angle coverage (°)	-51 to + 60 (dose symmetric)	-49 to + 59 (dose symmetric)	-60 to + 48 (dose symmetric)
Tilt series increment angle (°)	3	3	3
Electron dose per tilt image (e^- /Å^2)	3.01899	3.2152	2.15686
Electron dose per tomogram (e^- /Å^2)	114	120	80
